# Pneumatosis Intestinalis, Pneumoperitoneum, Pneumoretroperitoneum, Pneumomediastinum, and Pneumobilia After Pembrolizumab Therapy: A Case Report

**DOI:** 10.7759/cureus.55972

**Published:** 2024-03-11

**Authors:** Charles D Calenda, Cameron R Toohey, Madeline Levy, AyJy Bhardwaj, Jaspreet Ubhi, Arunima Sharma, Fady Abou Rizk

**Affiliations:** 1 Medicine, College of Osteopathic Medicine, University of New England, Biddeford, USA; 2 General Surgery, Concord Hospital - Laconia, Laconia, USA; 3 Internal Medicine, Concord Hospital - Laconia, Laconia, USA; 4 Critical Care, Concord Hospital - Laconia, Laconia, USA

**Keywords:** immune checkpoint inhibitor (ici), unexpected events, pneumatosis intestinalis, unusual combination of conditions, treatment or management challenges, rare disease or condition

## Abstract

Immune checkpoint inhibitors (ICIs) are becoming increasingly popular in treating cancers resistant to traditional chemotherapy. While ICIs have shown promise in treating cancer, the class of drugs also comes with certain risks, such as the development of pneumatosis intestinalis (PI) in rare cases. Pembrolizumab, an ICI that inhibits programmed cell death protein 1 (PD-1), has, in some rare instances, caused PI. Patients with ICI-induced PI may also present with pneumoperitoneum, pneumoretroperitoneum, pneumomediastinum, and pneumobilia. In the current report, we describe the presentation and management of a 50-year-old female with initial complaints of diffuse abdominal pain, constipation, abdominal distension, nausea, and decreased urine output approximately six months after beginning pembrolizumab and two months after the most recent dose of pembrolizumab. Subsequent CT imaging revealed massive PI with pneumoperitoneum, pneumoretroperitoneum, pneumomediastinum, and pneumobilia suspected to be secondary to pembrolizumab. Here, we discuss the possible mechanisms of ICI-induced PI and evaluate the management of patients presenting with PI and pneumoperitoneum.

## Introduction

Pneumatosis intestinalis (PI) is a rare event secondary to multiple etiologies in which air extravasates into the intestinal wall, with a mortality rate of 22% [1]. Approximately 15% of PI cases are idiopathic, while 85% are secondary [2]. In a population study characterizing different secondary causes of PI, Treyaud et al. showed that the majority of PI cases were due to intestinal ischemia (53.7%), obstructive etiologies (8.1%), non-obstructive etiologies (6.7%), and medications (5.4%) [3]. Medications remain an infrequent cause of PI, yet pose a clinical challenge to patients and their clinicians, as some anticancer drugs have been associated with a delayed presentation and recurrence of PI after re-administration of the offending agent [4]. Gazzaniga et al. found that 25% of anticancer drug-induced PI occurred 12 weeks after starting treatment, and 33% of patients who received the same anticancer medication redeveloped PI [4].

Identifying a medication as the causative factor is essential considering the recurrence rate, potential delay in presentation, and overall high mortality rate associated with PI. More recently, immune checkpoint inhibitors (ICIs), an emerging class of anticancer drugs typically reserved for treating resistant cancer, have been associated with PI rarely [5]. There are limited studies regarding the implication of pembrolizumab, a programmed cell death protein 1 (PD-1) inhibitor, in developing PI [4-7]. Here, we present an unusual case of a 50-year-old female patient previously treated with pembrolizumab for the recurrence of triple-negative breast cancer. Approximately six months after initiating pembrolizumab and two months after the most recent treatment, the patient developed massive PI with pneumoperitoneum, pneumoretroperitoneum, pneumomediastinum, and pneumobilia.

## Case presentation

Our patient is a 50-year-old white female with a weight of 85 kilograms and a BMI of 30 who presented to the emergency department (ED) with complaints of constipation for three days, decreased urine output, diffuse abdominal pain rated 8/10, nausea, and abdominal distension. Pertinent past medical history included breast cancer status post lumpectomy and chemotherapy, triple-negative recurrence status post immune checkpoint inhibitor therapy, drug-induced pneumonitis, pulmonary hypertension, sleep apnea with continuous positive airway pressure use, cirrhosis, tobacco use, and emphysema. Her pulmonary history was notable for moderate to severe emphysematous changes seen on CT noted as early as five years prior. The patient did not report coughing or wheezing at this presentation, indicating that her emphysema was well controlled with ipratropium bromide/albuterol, albuterol, and budesonide-formoterol.

Doxorubicin, cyclophosphamide, and paclitaxel were administered three years prior for triple-negative breast cancer with a positive response. Pembrolizumab and nab-paclitaxel were started six months before this presentation after the recurrence of triple-negative breast cancer. The patient received the most recent doses of pembrolizumab and nab-paclitaxel approximately two months prior to presentation. Methadone (80 mg/day) was started two months before presentation for opioid use disorder. Lactulose (10 g/15 mL, 5x/day) was used to reduce constipation from methadone therapy.

Vital signs were within normal limits when the patient presented to the ED. The patient's oxygen saturation level was 95% on room air. Physical examination revealed a chronically ill-appearing female with a distended abdomen tender to palpation throughout with guarding. Respirations were even and unlabored, and the lungs were clear to auscultation bilaterally. Labs were largely unremarkable, except for subtle irregularities, including mildly elevated white blood cell count (10.4 x 10^9^/L), mild thrombocytopenia (141 x 10^9^/L), hypoalbuminemia (3.2 g/dL), and hypokalemia (3.3 mmol/L).

**Figure 1:**
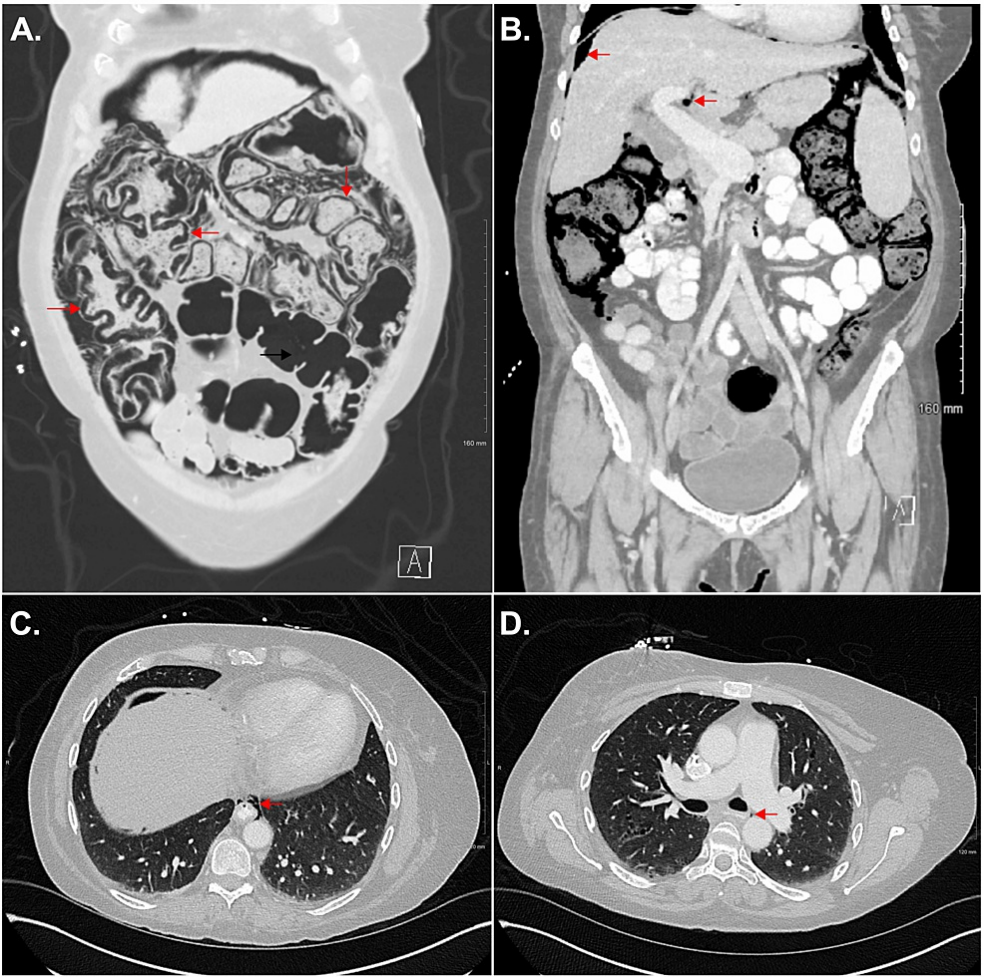
Transverse and coronal CT images showing pneumatosis intestinalis, pneumobilia, pneumomediastinum, and pneumoperitoneum (A) Coronal CT showing massive pneumatosis intestinalis (PI) in the ascending, transverse, and descending colon. Note the absence of PI in the sigmoid colon (represented by a single black arrow). (B) Coronal CT showing pneumoperitoneum (superior red arrow) and pneumobilia (inferior red arrow). (C) Transverse CT image showing pneumomediastinum at the level of the diaphragm (red arrow). (D) Transverse CT image showing pneumomediastinum less prominent more superiorly, just below the level of the carina (red arrow).

CT revealed massive PI throughout the ascending, transverse, and descending colons, marked colonic distention, and abnormalities at the sigmoid colon area indicating possible colonic perforation (Figure 1). Pneumoperitoneum, pneumoretroperitoneum, a small amount of discontinuous pneumomediastinum, pneumobilia, and significant constipation were also present (Figure 1). In the ED, the patient received piperacillin-tazobactam (4.5 g/100 mL), cefazolin (2 g/100 mL), and a bolus of sodium chloride 0.9% (1000 mL). The patient was made nil per os (NPO) and was scheduled for exploratory laparotomy to assess for colonic perforation, indicated due to the patient's peritoneal signs and CT findings concerning perforation.

During exploratory laparotomy on day one of hospitalization, the peritoneal cavity was entered, followed by an immediate gust of air compatible with pneumoperitoneum. Colonic dilatation and marked pneumatosis of the colon were noted, extending from the cecum to the proximal descending colon. Extensive gas was present within the omentum and mesentery. No injury, perforation, volvulus, or signs of ischemia were noted from the ligament of Treitz to the terminal ileum and throughout the colon. The colon was decompressed manually from the cecum to the proximal sigmoid. Superior extension of the incision revealed unremarkable findings of the stomach and duodenum.

The abdominal wound was kept open for the second day of hospitalization for a second look exploratory laparotomy. It was noted that there was an improvement in the dilation of the colon and, again, no signs of perforation. The abdominal wall was closed following the placement of a BLAKE® drain (Ethicon Endo-Surgery Inc., Cincinnati, USA). Critical care medicine was consulted since the patient remained intubated post-operatively for ventilatory support. An IV fentanyl drip (1000 mcg/100 mL) was initiated in place of oral methadone (80 mg/day) for opioid use disorder as the patient was NPO.

Clinical improvement was noted throughout the hospital stay, including reduced abdominal pain and distension. She was able to pass flatus on the fourth day of admission. A liquid diet with bisacodyl was initiated on the sixth day of admission. The patient was able to tolerate solids and have a bowel movement on the seventh day. She had significant improvements in abdominal distension and pain and was subsequently discharged.

## Discussion

The current study highlights an unusual presentation of massive PI with additional findings of pneumoperitoneum, pneumoretroperitoneum, pneumomediastinum, and pneumobilia, suspected to be secondary to pembrolizumab. The most common causes of PI, including intestinal ischemia, obstructive etiologies, and non-obstructive etiologies [3], were ruled unlikely based on the patient's pertinent past medical history, CT imaging, and exploratory laparotomies. We evaluate multiple potential causes of our patient's PI here, including pembrolizumab, nab-paclitaxel, and emphysema. While the current study does not include histological findings, future studies should report if histology is obtained to characterize the pathophysiology underlying pembrolizumab-induced PI, which may help guide the management of these patients moving forward.

Together, the time to PI onset after initiating pembrolizumab and an increase in suspected pembrolizumab-induced PI cases [4-7] support pembrolizumab as the most likely catalyst here. Sperling et al. found that in patients who developed suspected cases of ICI-induced PI, the median time to onset after initiating the drug was seven months and occurred within one year of the most recent dose [5]. The onset from initiation and most recent dose in our case closely aligns with the findings from Sperling et al. [5], seeing that our patient presented approximately six months after starting pembrolizumab and two months after her most recent dose. A delay in adverse effects of ICIs seen here and in other studies may be explained by several hypothesized mechanisms, including a latent or subclinical autoimmune state induced by ICIs [8]. Increased gut permeability is a proposed mechanism in which PI can develop in the setting of normal intraluminal pressures, among other causes [9]. While no precise mechanism has been identified as the catalyst in ICI-induced PI, it has been speculated that ICIs may increase gut permeability through ICI-mediated inflammation and tissue damage in other gastrointestinal pathologies [10]. Histological findings in future studies similar to our case may help determine if increased gut permeability is an underlying mechanism of ICI-induced PI.

Paclitaxel, a medication our patient received for the treatment of her triple-negative breast cancer, has been implicated in PI [4,11-12]. Approximately four years ago, our patient received four cycles of paclitaxel for triple-negative breast cancer. After the recurrence of the patient's triple negative breast cancer was found this year, she received the albumin-bound form of paclitaxel, nab-paclitaxel, which has been shown to cause fewer side effects than the unbound form of paclitaxel [13]. To the best of our knowledge, there are no reports of nab-paclitaxel causing PI. Considering her distant exposure to paclitaxel and the use of the safer form of paclitaxel, nab-paclitaxel, more recently, it is doubtful that either caused PI here.

Obstructive lung diseases have been implicated in PI [14], and our patient's history was significant for emphysema. A proposed mechanism in which obstructive lung disease can lead to PI is through alveolar rupture of air tracking down the mediastinal vessels and into the intestinal wall [9]. More recently, the lack of interstitial involvement in some pulmonary cases has cast doubt on this theory [9]. Another proposed mechanism is that transient increases in intraabdominal pressure from coughing can lead to extravasation of air into the intestinal wall, ultimately leading to PI [9]. In our case, emphysema is an unlikely cause of PI based on the patient denying coughing and clinical findings supporting that her emphysema was well controlled. During the current presentation, the patient showed no clinical signs, such as wheezing or prolonged expiration, on physical examination in the ED and upon admission to the hospital. Additionally, the pneumomediastinum was undoubtedly secondary to the retroperitoneal gas extending through the hiatus. The massive PI involving all three segments of the colon seen in our patient (Figure 1) was unlikely to have been produced by air traveling down the mediastinum, considering the small amount of air seen in the mediastinum.

Regarding the patient's CT finding of pneumoperitoneum with PI, pneumoperitoneum appears to be a common finding in patients with ICI-induced PI. Sperling et al. found that pneumoperitoneum was present in 50% of patients with ICI-induced PI [5]. In the current case, the pneumoperitoneum was likely secondary to massive ICI-induced PI, given the lack of perforation at surgery. While it is worth noting that the pneumomediastinum seen on CT in our patient contained only a small amount of air, the presence of air in the mediastinum, peritoneum, retroperitoneal space, and biliary tree truly underscores the extent of involvement of our patient's PI.

Depending on the clinical scenario, a diagnosis of PI can be managed conservatively or through surgical intervention. Conservative treatment generally involves nasogastric bowel decompression, parenteral nutrition, and prophylactic antibiotics [15]. A retrospective study by Morris et al. found that up to 50% of patients with PI were managed conservatively with successful outcomes [1]. Notably, patients in the operative group were found to have a higher mortality of 16% compared to the non-operative group's mortality of 6% [1].

Other cases of PI associated with ICI therapy have been successfully managed with conservative treatment [5-6]. Khalil et al. previously developed an algorithm for determining whether emergent surgery, re-assessment, or a conservative approach would be indicated in patients with PI [16]. Indications for a surgical approach in the algorithm include concomitant critical CT findings, conspicuous physical examination, conspicuous physical condition, and critical laboratory findings [16]. In the current case, a conservative approach would have been unsuitable based on critical CT findings suggestive of a perforation and peritoneal signs on physical examination. Critical laboratory findings, also in the algorithm discussed by Khalil et al. [16], were absent in our patient. However, laboratory findings must be assessed with caution in immunocompromised and chronically ill cancer patients, both of which were pertinent to our patient's history.

In select surgical scenarios with suspected perforation, exploratory laparotomy or diagnostic laparoscopy may be performed based on surgical judgment. Exploratory laparotomy was chosen in this case because the approach saved time, offered better exposure, and allowed manual palpation to evaluate for possible intraluminal tumors. The patient was chronically ill and had been hospitalized multiple times over the past year, with no recent colonoscopy. Additionally, there was marked dilatation of almost the entire colon seen on CT (Figure 1). Considering the marked dilatation, it would be challenging to examine the entire gastrointestinal tract to identify a site of perforation or tumor laparoscopically. With the colonic walls being distended and friable, instrumental manipulation could result in iatrogenic perforation. For our patient, we found that exploratory laparotomy was effective and safe to rule out suspected colonic perforation or other pathologies.

## Conclusions

While extremely rare, suspected cases of pembrolizumab-induced PI are being reported more frequently in the literature. Future studies that further characterize pembrolizumab-induced PI and identify key histological findings may help guide the medical management of these patients. Clinicians should remain cognizant of PI as a rare potential adverse event from ICI treatment that frequently presents with pneumoperitoneum, but may present with air in multiple anatomical spaces, as seen in our patient. Surgeons and clinicians should be aware that in the proper clinical context in patients with PI, a conservative approach has retrospectively been associated with lower mortality rates compared to a surgical approach. Regardless, surgery may be indicated for clinical and diagnostic findings suggesting a more acute process in the presence of PI. In patients with PI involving marked dilatation of the colon with suspicion of perforation, surgery is indicated. Surgeons may weigh the risks and benefits of either diagnostic laparoscopy and/or laparotomy depending on the patient's clinical findings.

## References

[1] Morris MS, Gee AC, Cho SD, Limbaugh K, Underwood S, Ham B, Schreiber MA (2008). Management and outcome of pneumatosis intestinalis. Am J Surg.

[2] Goldberg E, Thomas Lamont MDJ (2024). Pneumatosis intestinalis. UpToDate.

[3] Treyaud MO, Duran R, Zins M, Knebel JF, Meuli RA, Schmidt S (2017). Clinical significance of pneumatosis intestinalis - correlation of MDCT-findings with treatment and outcome. Eur Radiol.

[4] Gazzaniga G, Villa F, Tosi F (2022). Pneumatosis intestinalis induced by anticancer treatment: a systematic review. Cancers (Basel).

[5] Sperling G, Shatila M, Varatharajalu K (2023). Pneumatosis intestinalis in cancer patients who received immune checkpoint inhibitors. J Cancer Res Clin Oncol.

[6] Yik B, Shah N (2021). Gas bubbles: a persistent problem with immunotherapy. Dig Dis Sci.

[7] Zhang T, Cao M, Zhao B (2023). Pneumatosis intestinalis post steroid use in a patient with immune-related adverse events: case report, literature review and FAERS analysis. Front Pharmacol.

[8] Cheng Y, Ling F, Li J, Chen Y, Xu M, Li S, Zhu L (2023). An updated review of gastrointestinal toxicity induced by PD-1 inhibitors: from mechanisms to management. Front Immunol.

[9] St Peter SD, Abbas MA, Kelly KA (2003). The spectrum of pneumatosis intestinalis. Arch Surg.

[10] Pezo RC, Wong M, Martin A (2019). Impact of the gut microbiota on immune checkpoint inhibitor-associated toxicities. Therap Adv Gastroenterol.

[11] Guiu S, Ortega-Deballon P, Guiu B (2011). Pneumatosis intestinalis and pneumoperitoneum during treatment by paclitaxel. Surgery.

[12] Brocchi S, Parmeggiani A, Gaudiano C (2021). Pneumatosis intestinalis and spontaneous perforation associated with drug toxicity in oncologic patients: a case series. Acta Gastroenterol Belg.

[13] Mahtani RL, Parisi M, Glück S (2018). Comparative effectiveness of early-line nab-paclitaxel vs. paclitaxel in patients with metastatic breast cancer: a US community-based real-world analysis. Cancer Manag Res.

[14] Im J, Anjum F (2023). Pneumatosis intestinalis. StatPearls [Internet].

[15] Al-Talib A, Al-Ghtani F, Munk R (2009). Pneumatosis intestinalis: can we avoid surgical intervention in nonsurgical patients?. Case Rep Gastroenterol.

[16] Khalil PN, Huber-Wagner S, Ladurner R (2009). Natural history, clinical pattern, and surgical considerations of pneumatosis intestinalis. Eur J Med Res.

